# Modelling the Spatio-Temporal Cell Dynamics Reveals Novel Insights on Cell Differentiation and Proliferation in the Small Intestinal Crypt

**DOI:** 10.1371/journal.pone.0037115

**Published:** 2012-05-18

**Authors:** Carmen Pin, Alastair J. M. Watson, Simon R. Carding

**Affiliations:** 1 Integrated Biology of the Gastrointestinal Tract, Institute of Food Research, Norwich, United Kingdom; 2 Norwich Medical School, University of East Anglia, Norwich, United Kingdom; University Claude Bernard Lyon 1, France

## Abstract

We developed a slow structural relaxation model to describe cellular dynamics in the crypt of the mouse small intestine. Cells are arranged in a three dimensional spiral the size of which dynamically changes according to cell production demands of adjacent villi. Cell differentiation and proliferation is regulated through Wnt and Notch signals, the strength of which depends on the local cell composition. The highest level of Wnt activity is associated with maintaining equipotent stem cells (SC), Paneth cells and common goblet-Paneth cell progenitors (CGPCPs) intermingling at the crypt bottom. Low levels of Wnt signalling area are associated with stem cells giving rise to secretory cells (CGPCPs, enteroendocrine or Tuft cells) and proliferative absorptive progenitors. Deciding between these two fates, secretory and stem/absorptive cells, depends on Notch signalling. Our model predicts that Notch signalling inhibits secretory fate if more than 50% of cells they are in contact with belong to the secretory lineage. CGPCPs under high Wnt signalling will differentiate into Paneth cells while those migrating out from the crypt bottom differentiate into goblet cells. We have assumed that mature Paneth cells migrating upwards undergo anoikis. Structural relaxation explains the localisation of Paneth cells to the crypt bottom in the absence of active forces. The predicted crypt generation time from one SC is 4–5 days with 10–12 days needed to reach a structural steady state. Our predictions are consistent with experimental observations made under altered Wnt and Notch signalling. Mutations affecting stem cells located at the crypt floor have a 50% chance of being propagated throughout the crypt while mutations in cells above are rarely propagated. The predicted recovery time of an injured crypt losing half of its cells is approximately 2 days.

## Introduction

In the small intestine the boundary layer of epithelial cells is folded to form a number of invaginations or crypts. Epithelial renewal is driven by stem cells located at the bottom of the intestinal crypt. Epithelial cells produced in the lower part of the crypt progressively migrate upward simultaneously proliferating and differentiating into absorptive enterocytes or secretory cells such as mucus-secreting goblet cells, enteroendocrine cells or recently identified Tuft cells, which release opioids through an exocrine–paracrine mechanism [1]. An additional differentiated secretory cell, the Paneth cell, populates the crypt base [2].

Historically intestinal stem cells were identified by their long-term retention of labelled DNA, their location above the Paneth cell compartment at a position four cells distant from the crypt base [3], and preferential expression of various transcriptional factors (e.g. Musashi-1 and Hes1) [4]. Now, expression of the leucine-rich repeat-containing G-protein coupled receptor 5, Lgr5, is considered the definitive stem cell marker [5] with individual Lgr5^hi^ cells from the crypt base being capable of forming self-organizing crypt–villus organoids containing all epithelial cell lineages [6], [7]. Another region of the crypt at position +4 has also been shown to contain stem cells capable of giving rise to all the small intestinal cell lineages [8]. These cells are slow cycling or quiescent stem cells and express the homeobox genes *Bmi1* and *Hopx*, express telomerase reverse transcriptase, and give rise to mitotically active, *Lgr5* expressing stem cells that reside in stem cell niche in between Paneth cells [9], [10], [11], [12]. The co-expression of the marker genes *Lgr5*, *Bmi1* and *mTert* has been reported overlapping in cells located in the crypt base [13], [14]. It is not clear if stem cells in position +4 and at the crypt base are distinct, overlapping or identical stem cell populations [12], [15].

The dynamics of Paneth cell development remains unclear. They are the only cell type reported to migrate downward to the crypt bottom as a result of repulsive forces generated by the tyrosine kinase guidance receptors and T cell factor (TCF) targets EpHB2 and EphB3, and the Frizzled-5 receptor [2]. Conditional deletion of these receptors or their ligands results in aberrant Paneth cells with altered distribution along crypts and villi [16], [17], [18]. However, the presence of intermingling proliferating cells amongst Paneth cells [19] whose descendents migrate upwards complicates the understanding of the downward migration of Paneth cells.

The Wnt and Notch signalling pathways are key mediators and regulators of stem cell proliferation and their differentiation into absorptive and secretory lineages [2]. The highest levels of Wnt signalling are observed in cells located at the crypt bottom decreasing gradually along the crypt villus axis [20]. Notch receptors regulate a large spectrum of cell fate decisions [21] and recently Notch signalling has been shown to be activated in intestinal stem cells with expression of Notch ligands being required for homeostasis [22], [23].

In the study reported here we have used a Montecarlo model to describe the intestinal crypt in a slow structural relaxation regime which omits the active individual forces described in other individual cell based models [24], [25], [26], [27]. Although slow dynamics is frequently identified in biological systems such as tissues, bacterial colonies or high-density cultures, to our knowledge this is the first time this approach has been used to model the intestinal crypt. In addition, the model excludes an explicit external structural scaffold with cells arranged in a three dimensional spiral formed by flexible rings of cells the size of which can be dynamically changed to adjust to the demands on cell production by adjacent villi. Cellular proliferation and differentiation into secretory and absorptive cellular lineages are determined by Wnt and Notch signals that are modelled as a function of the local cell composition, which contrasts with existing models in which Wnt activity is modelled as the response to a gradient of extracellular Wnt factors along the crypt axis [24] or, is associated with the local curvature of the external scaffold or basal membrane containing the crypt [26] with Notch activation depending on threshold parameters that differ according to the cell type [26].

Our model of intestinal crypt dynamics identifies feasible mechanisms of cell differentiation and proliferation evaluated by comparing predictions with available empirical observations. The model also creates a simulation framework for crypt development and homeostasis, for the generation of robust predictions on the effect of altering signalling pathways, the propagation of mutations throughout the crypt and, on the recovery times after crypt injury.

## Results

### Model Hypotheses Underlying Cell Proliferation and Differentiation in the Crypt

**Figure 1:**
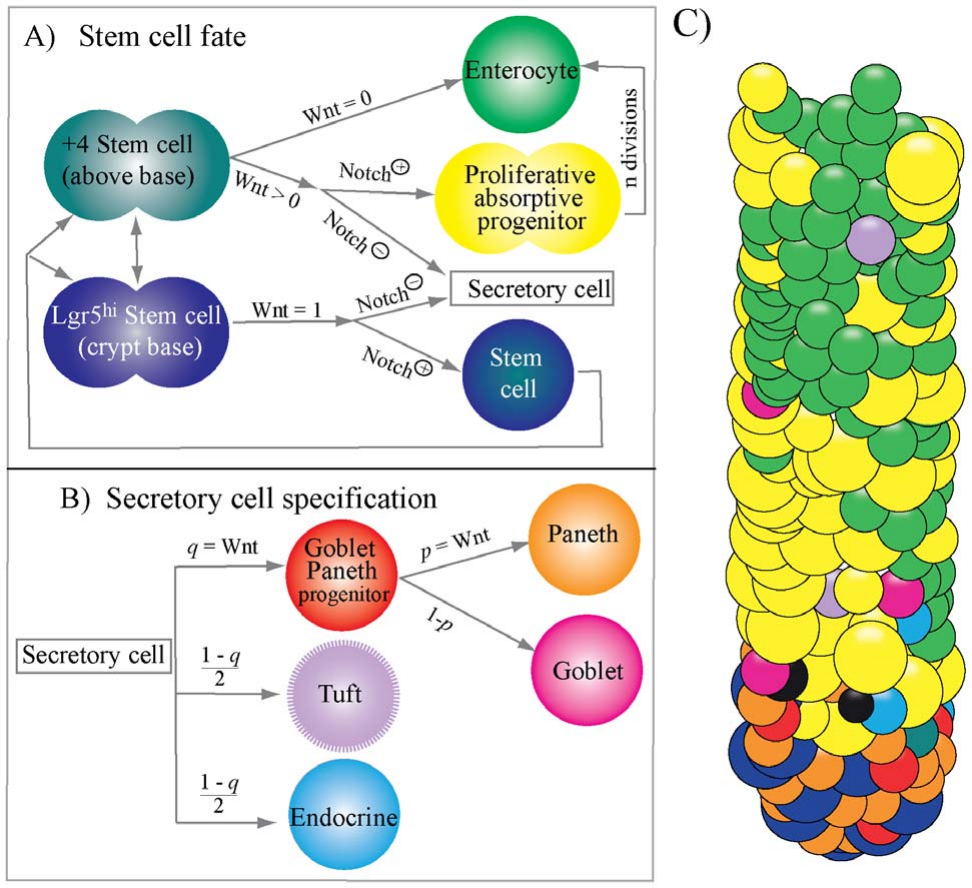
Model for cellular differentiation and proliferation in the crypt of mouse small intestine. A) Fate of stem cells progeny. Stem cells at the crypt bottom and at position +4 replenish each other and give rise to different progeny according to the strength of Wnt signalling. Wnt signalling is quantified according to the local cell composition as described in Equation 1 with a maximum value of 1 in the crypt base. Under the highest levels of Wnt signalling at the crypt bottom (Wnt  = 1), stem cells progeny consists of secretory cells and stem cells. At lower levels of Wnt signalling (Wnt <0) stem cells give rise to secretory cells and proliferative absorptive progenitors. In both cases the decision between these two fates, secretory and stem/absorptive, depends on Notch signalling. Differentiation into the secretory cellular lineage is inhibited if Notch is activated (Notch^⊕^) as a result of 50% or more of adjacent cells belonging to the secretory lineage. B) Secretory cell specification. Secretory cells differentiate into goblet Paneth cell common progenitors with a probability estimated as *q*  =  Wnt signalling (quantified from Equation 1), otherwise secretory cells become either Tuft or enteroendocrine cells with equal probability. Goblet-Paneth cell common progenitors differentiate into Paneth cells with probability *p*  =  Wnt signalling (Equation 1), otherwise they differentiate into goblet cells. C) A snapshot of the simulated crypt in steady state shows how all cell types locate on the three dimensional spiral.

Asymmetric crypt and villus structures containing heterogeneous cell types are generated under uniform extracellular signalling environments. Therefore, differential responsiveness to signalling rather than differential exposure to extracellular signal governs cell proliferation and differentiation in the crypts [6]. We hypothesised that signals and responses are determined by interactions between neighbouring cells and coupled with cell lineage commitment. Figure 1 summarises our hypotheses to model cell differentiation and proliferation.

Empirical observations demonstrate that only Paneth and stem cells are located in the crypt bottom cap where the highest levels of Wnt signals are expressed [2]. In our model, we have assumed that stem cells in the crypt base that are exposed to the highest levels of Wnt signalling give rise to stem cells and secretory cells. The size of the crypt base or crypt bottom cap is determined according to the crypt dimensions (see Methods section). By contrast, stem cells located above the crypt base or exposed to low Wnt stimuli generate proliferative progenitors of absorptive epithelial cells and secretory cells (Fig. 1). The decision as to whether stem cells give rise to secretory or to stem/absorptive lineages depends on Notch signalling.

#### 1) Wnt signalling

We have assumed that cells generated in the crypt bottom cap under the influence of the highest level of Wnt signalling are committed to becoming either stem cells or common goblet-Paneth cell progenitors and that these cell lineages simultaneously generate high Wnt signalling environments. Published results support the production of Wnt signals by Paneth cells and proliferative cells [19], [28]. Wnt signalling is quantified as follows:

For any cell in the crypt, Wnt signalling is modelled according to the local cell composition as:
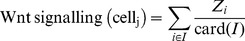
(1)Where



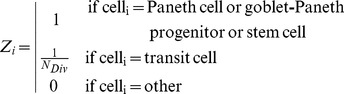
(2)
*N_Div_* is the number of divisions already undertaken by proliferative absorptive progenitors; *I* is the set of cells in the rings located immediately above and below the cell in question, cell_j_; card(*I*) is the number of cells in those rings.

**Figure 2:**
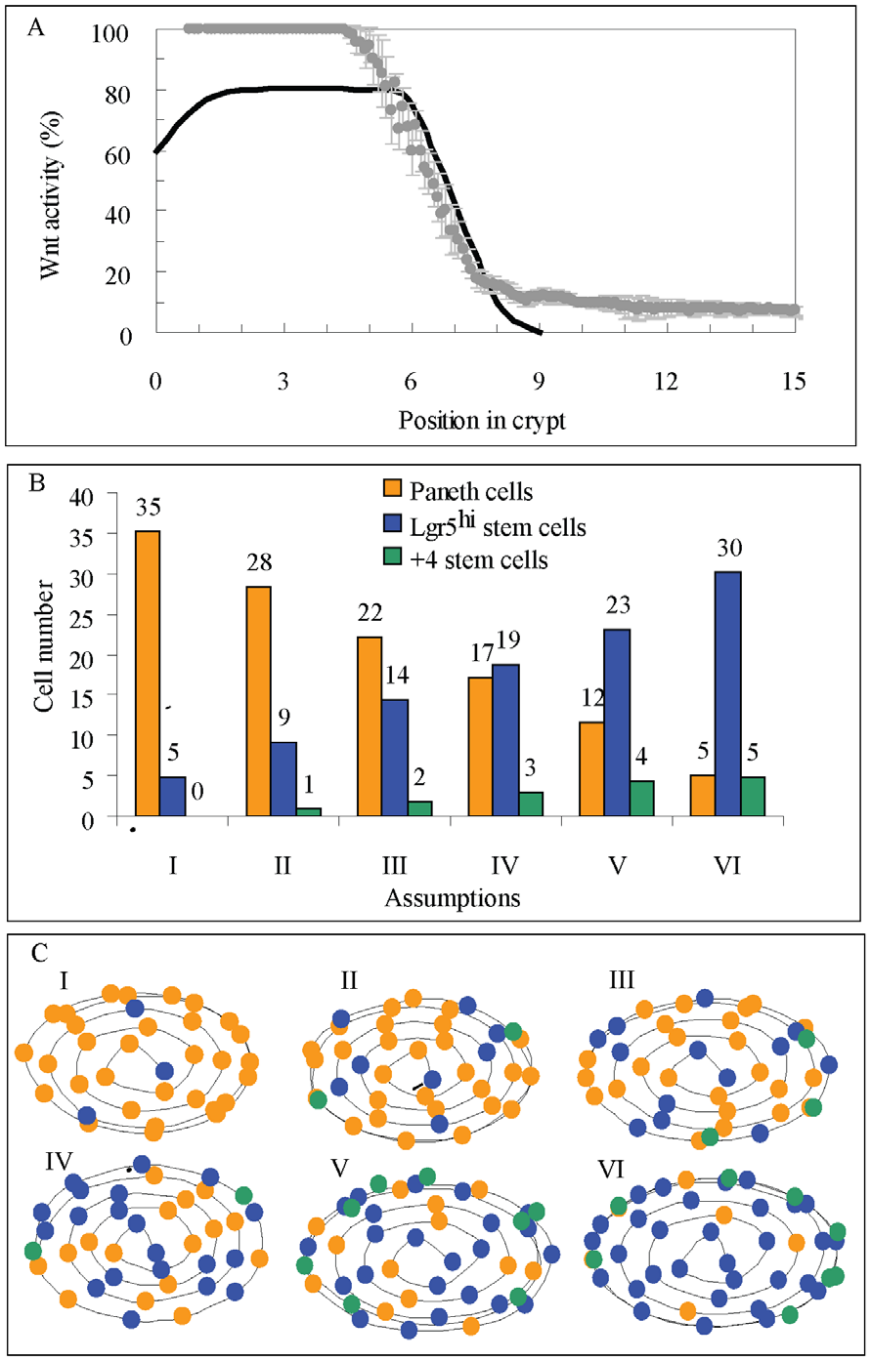
Modelling Wnt and Notch signalling in Paneth cells intermingled with Lgr5^hi^ stem cells at the crypt base. A) Wnt signalling activity (grey circles) as simulated by our model and measured experimentally (solid line) [28]. A Wnt activity level of 1 (Equation 1) is equivalent to 100%. The results shown were obtained from 30 simulated crypts. B) Number of Paneth or Paneth progenitor and stem cells at the bottom of the crypt under high Wnt signalling activity (100%) assuming that a newly generated cell differentiates into a common goblet Paneth cell progenitor if in contact with 1 or more stem cell; otherwise secretory fate is inhibited by Notch signalling (hypothesis I). Similarly, hypotheses II, III, IV, V and VI assume that newly generated cell differentiate into a Paneth cells if in contact with a number of stem cells equal or greater than 2, 3, 4, 5 and 6, respectively. The total number of cells in contact is 6. Hypothesis III, which states that secretory fate of a stem cell is inhibited by Notch signalling if it is in contact with more than 50% of cells that belong to the secretory lineage, which best fits empirical observations. The results shown are from 30 simulated crypts. Coefficient of variation for the number of cells (CV  =  standard deviation/mean) varied from 0.015 to 0.04 in all cases. C) Upper view of the distribution of Paneth and stem cells in the crypt base at day 20 according to the six hypotheses.

The Wnt signalling activity quantified for each cell varied from 0 to 1, with 0 reflecting cells that are not near Paneth and/or proliferative cells and 1 (or 100% in Fig. 2A) representing cells surrounded by Paneth and/or stem cells. From Equation 1, it follows that Wnt signalling is equal to 1 for all cells located in the crypt bottom (Fig. 1). A reduction in the levels of Wnt signalling is a consequence of the diminishing number or absence of Paneth and stem cells resulting in progressive cell differentiation as the cell ascends the crypt. These Wnt signalling predictions are consistent with independent published observations [19], [28] (Fig. 2A).

#### 2) Notch signalling

Notch signalling is tightly associated with binary cell fate decisions. Notch-signalling requires contact between cells and is activated in cells expressing Notch receptors that are juxtaposed with cells expressing notch ligands [29], [30]. Secretory cells are assumed to produce Notch ligands while activated Notch receptors have been reported in absorptive epithelial progenitors [26]. Recent studies have reported that Notch1 receptors are activated in intestinal stem cells [22] and that Notch signalling modulates proliferation and differentiation of intestinal stem cells at the crypt base [23]. In addition, physical contact with Paneth cells is essential for maintaining the stem cell niche [19], [31]. Therefore, based on these data we assumed that Paneth cells and their progenitors in the crypt bottom trigger Notch signalling in stem cells. In the following section, we predict that Notch signalling is activated in the stem cell progeny if more than 50% of cells they are in contact with belong to the secretory lineage. Activation of Notch signalling inhibits secretory cell fate with stem cells giving rise to either daughter stem cells, at the crypt base, or proliferative absorptive progenitors, above the crypt base.

#### 3) Paneth cells intermingling with equipotent Lgr5^hi^ stem cells in the crypt base

We tested several hypotheses to obtain the correct ratio of secretory cells required to trigger Notch signalling in order to obtain an intermingled distribution of Paneth and stem cells as experimentally observed [19], [31]. Figures 2B and C show the results of these simulations. In hypothesis I, a newly generated cell differentiates into a common goblet-Paneth cell progenitor if it contacts one or more stem cells, otherwise it remains a stem cell. Similarly, under hypotheses II, III, IV, V and VI newly generated cells differentiate into common goblet-Paneth cell progenitors if they are in contact with a number of stem cells equal or greater than 2, 3, 4, 5 and 6, respectively. Under hypothesis III, approximately 22 Paneth cells are intermingled with 14 Lgr5+^hi^ stem cells (Fig. 2B and C), in agreement with experimental observations [32] [31]. Hypothesis III was therefore selected for further use in the dynamic model of crypt development. A newly generated cell will differentiate into a secretory cell when in contact with 3 or more stem cells (i.e. more than 50% of cells in contact). Conversely the secretory fate is inhibited if more than 50% of the cells in contact with the stem cell belong to the secretory lineage (Fig. 1). At the crypt base under conditions of maximum levels of Wnt signalling, secretory cells become common progenitors for goblet and Paneth cells with a probability equal to one which estimated as the Wnt signalling quantified from equation 1 (Fig. 1). Thus, the probability of specification into other secretory types under the maximum level of Wnt signalling is 0.

It has been suggested that goblet and Paneth cells derive from a fate-restricted common progenitor [33]. Cells co-expressing markers of Paneth and goblet cells have been detected along villi following severe disturbance of intestinal epithelium homeostasis [34]. Experimentally, Wnt signalling has proven to be required for Paneth cell maturation [18]. In our model, common progenitor cells give rise to Paneth cells with a probability dependent upon the Wnt signalling stimulus in Equation 1, which is equal to 1 at the crypt base, otherwise common progenitors migrating upwards will differentiate into goblet cells (Fig. 1). We assumed that the maturation time of the common progenitor is the time required to reach the final cell size, which is shorter than a division cycle time as explained in Methods section that is supported by studies reporting that cells committed to the secretory lineage express markers of differentiated cells in less than a division cycle time [35].

Because of the intermingled distribution with proliferative cells, some mature Paneth cells will eventually migrate upwards and leave the bottom of the crypt. In the vast majority (95%) of crypts, the highest location of Paneth cells is below the +7 cell position from the crypt bottom [36]. Of more than 30,000 Paneth cells only 30 were found in the upper levels of the crypt with only 5 in the villus [32]. We assumed therefore that any Paneth cell migrating up the crypt body undergo cell death mediated by Eph/ephrin signalling, which controls cell compartmentalization of Paneth cells in the bottom of the crypt [16]. Shedding of E-cadherin molecules has been reported in the boundaries between compartmentalized populations of EphB- and ephrin-B expressing cells [37]. Thus, Paneth cells expressing EphB could detach when forced into a monolayer of absorptive progenitors expressing ephrin-B that restricts their adhesion. As the majority of apoptotic cells are located at the bottom of the crypt in the stem cell area, apoptosis has been assumed to maintain stem cell number and integrity [3], [38]. However, in the large intestine where crypts have no Paneth cells, apoptosis is a rare event and is not associated with the stem cell position [3]. Our model predicts that the bottom of the crypt contains approximately 2% apoptotic cells which is consistent with the levels (1–10%) detected in the healthy small intestinal epithelium of mice and humans [3].

#### 4) +4 stem cells, epithelial absorptive progenitors and secretory cells in the crypt body

In our model, +4 stem cells are the stem cells that migrate out of the crypt base to occupy the position 4 cells above the crypt base (Fig. 1). We assumed that both populations of stem cells at the base of the crypt and at the +4 position are part of the same stem cell pool, but give rise to different progeny due to differences in Wnt signalling. While stem cells at the crypt base give rise to stem cells and Paneth cells, the progeny of +4 stem cells will specify into proliferative absorptive progenitors if more than 50% of the cells they contact belong to the secretory lineage, i.e. Notch is activated, otherwise the progeny of +4 stem cells will specify into secretory cells.

It has been shown that Notch signalling determines stem cell fate and the generation of either absorptive or secretory lineage via the transcription factor Atoh1 [39]. There is however some controversy as to whether Notch inhibition results in increased goblet cell production and whether the expansion of other secretory cell compartments is influenced [22], [23], [40], [41]. In accordance with the most recent experimental results [23], we assumed that Notch affects the differentiation of all four secretory cell lineages. Further specification into each secretory lineage is based on the fact that downstream of the transcription factor Atoh1, goblet and Paneth cells have similar transcription factor requirements for their differentiation which in turn are distinct from those involved in Tuft or enteroendocrine cell development [1]. Thus in our model, if secretory cell fate is not inhibited the progeny of +4 stem cells differentiate into common goblet-Paneth cell progenitors with a probability equal to the estimated Wnt signalling from Equation 1, which mirrors the fate of the stem cell secretory progeny at the crypt base. In the event of no specification into goblet-Paneth cells, secretory cells will differentiate into either enteroendocrine or Tuft cells with equal probability (Fig. 1).

We have hypothesised that goblet, Tuft and enteroendocrine cells are produced only above the base of the crypt as first descendents from stem cells and after their commitment to the secretory fate their proliferation is halted. This generates a decreasing number of goblet, Tuft and enteroendocrine cells ascending towards the top of the crypt. Consistent with our predictions, the maximum numbers of goblet and enteroendocrine cells are found in the basal portion of the crypt with a progressive decrease towards the upper part of the crypt [32], [36], [42], [43]. Recent observations support our model hypothesis of no proliferation among secretory cell progenitors. The detection of the Notch ligand Delta 1, which is a specific marker of secretory cells, has been reported to coincide with a block in further divisions and to be followed in less than one division cycle by the expression of typical markers of mature goblet, enteroendocrine and Paneth cells [35]. In the simulated crypt about 4% of cells generated are goblet cells, while enteroendocrine or Tuft cells make up ∼1% (Fig. S1A), consistent with empirical measurements [1], [2].

Absorptive epithelial progenitor cells are committed to undergo a number of divisions and to differentiate into absorptive enterocytes. Proliferative epithelial progenitors have been reported to have a limited self-renewal capacity [15] and the number of divisions they undertake has been estimated as described in the Methods section. Mature enterocytes and absorptive epithelial proliferating progenitors are abundant in the upper portion of the simulated crypt (Fig. S1A). The proportion of proliferating cells predicted at different positions in the crypt is consistent with published data [26] (Fig. S1B).

### Slow Dynamics and Cell Motion

We have used a Montecarlo model to describe crypt reorganization as a slow structural relaxation phenomenon. At each interval time of the simulation, the three dimensional spiral and helix are re-built according to the size of the cells and division or deletion events. Growing cells expand homogenously in all directions. The size of the rings of the spiral changes dynamically to accommodate cell size increases in the horizontal direction, perpendicular to the crypt-villus axis (Fig. S2A). The increase in cell size in the vertical direction creates a force translated mostly in upward migration. Figure S2B shows that the predicted ascending velocities are close to zero at the crypt bottom but increase progressively from position +4 to 12 where cells migrate at a velocity of ∼1 cell diameter per hour. These results agree with empirically determined migration velocities [3]. Thus, the force derived from cell growth is sufficient to explain cell dynamics in the crypt. The trajectories of Paneth cells that remain at the bottom of the crypt, and those of stem cells located at the crypt bottom, the progeny of which migrate upwards and differentiate into secretory and absorptive progenitors, are shown in Figure S2C. For cells located beyond position +7 in the crypt the model generates vertical trajectories towards the villi.

Our approach excludes active forces to account for the downward migration of goblet/Paneth progenitors and/or mature Paneth cells to the crypt bottom. The localisation of Paneth cells or their progenitors intermingling with stem cells at the crypt bottom is driven by Notch signalling as described above (Fig. 2C), and by the fact that Paneth cells are non-proliferating and therefore non-migratory cells. It can be demonstrated that in a mixed system of proliferating and non-proliferating cells restricted to the bottom of a cylindrical shape, active forces are not required to confine non-proliferating cells to the bottom of the cylinder. Actively dividing cells will eventually gain positions above non-proliferating cells (see Fig. S3).

### Modelling Crypt Generation from One Stem Cell

The final number of cells in a crypt is the only input parameter needed to build the crypt from a single stem cell. The length and diameter of the crypt and the size of the crypt bottom cap is estimated from the final number of cells. Relationships between the number of cells and crypt morphometric measurements were estimated from published data [32], [44] (see Methods section).

**Figure 3:**
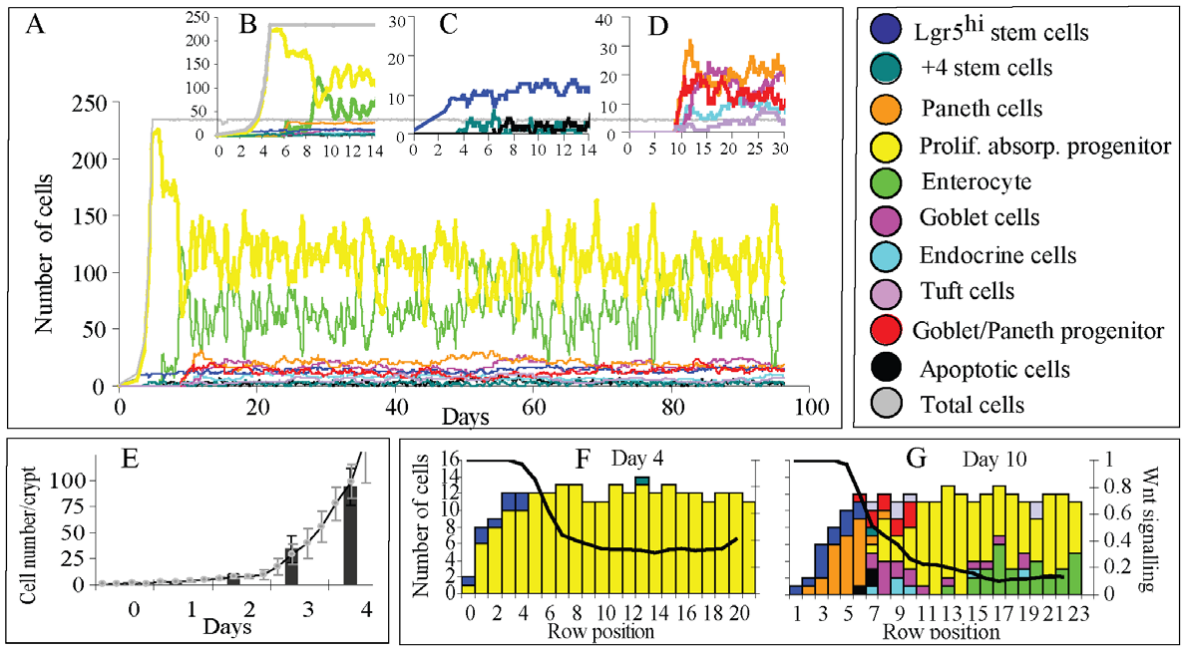
Modelling crypt development from a single stem cell. A–D) The number of cells during the development of a crypt from a single stem cell. The total number of cells, 235, is reached in about 5 days with a structural steady state reached by day 11. The simulation is for 100 days. E) Comparison between simulated (circles) and observed (columns) numbers of cells in a crypt developing from one stem cell [6]. Data shown is from 30 simulated crypts. F) Cellular crypt composition and Wnt signalling by day 4 when the growing crypt reaches its final number of cells. G) Cellular crypt composition and Wnt signalling by day 10 when the crypt reaches a structural steady state.

The crypts are initially modelled as closed spheroids with a central lumen that expand radially through cell proliferation. By 4–5 days when the crypt reaches its final size of 235 cells (Fig. 3A–D), the spheroid shape progressively elongates to form a large open-ended cylinder. Of interest, individual stem cells from the small intestine or colon initially give rise to closed spheroid-like organoids that then undergo multiple crypt fission events to generate villus like epithelial domains when grown in culture [6], [45]. Initially, only proliferative cells are generated during the expansion phase of the crypt with mature epithelial cells being generated after the crypt reaches its final size (Fig. 3A–D). The predicted number of cells during crypt expansion is in agreement with that observed in cultured organoids generated from a single cell [6] (Fig. 3E). There is a lack of data regarding the timing and order of appearance of differentiated cell types. We have therefore assumed that differentiation starts simultaneously for all cell lineages, once absorptive epithelial proliferating progenitors complete their committed cellular divisions and the first enterocytes are generated (Fig. 3B and D). It has been reported that all cell lineages are observed in cultured crypts within two weeks of seeding with single cells [6]. In our simulated crypts, cell differentiation starts by day 6, with a steady state structure obtained after day 9–10 (Fig. 3 F and G). A video of the generation of a crypt from a single stem cell and its development over 40 days is included in the supplementary material (Video S1).

### Clonal Evolution of Equipotent Lgr5^hi^ Stem Cells is Consistent with Neutral Drift Dynamics

Asymmetric DNA segregation during stem cell division is regarded as a protective DNA mechanism in which stem cells undergoing mitosis retain the original DNA strand and transfer the newly synthesised strand to their descendents [46], [47]. However, the random segregation of chromosomes in intestinal stem cells has been recently confirmed by genetic lineage-tracing experiments [48]. In addition clonal expansion has been used to demonstrate that stem cells are equipotent and divide symmetrical symmetrically following a neutral drift dynamics in which clones are lost or expand at random until one takes over the crypt [31], [49]. In our model stem cells are equipotent but do not always divide symmetrically. The cellular fate is decided according to neighbouring cells. With our hypothesis we estimate that 53% of the stem cells divisions are symmetric and 43% are asymmetric giving a stem cell and goblet-Paneth cell progenitor with the remaining 4% producing common goblet-Paneth cell progenitors. To demonstrate that our model is consistent with empirically determined neutral drift dynamics [31], we followed the clonal evolution of 14 equipotent Lgr5^hi^ stem cells in a simulated crypt comprising all cell lineages (Video S2). We observed rapid clonal expansion during the first 7–10 days (Fig. S4A–B) during which the number of clones was reduced from 14 to 2–3. Analysis of clonal evolution during the first week was consistent with a pattern of neutral drift dynamics. We also observed that the cumulative clone size distribution converged rapidly into scaling behaviour, characteristic of equipotent stem cells dividing symmetrically [31], [49] (Fig. S4C). The scaling function was estimated as previously described [31] using a cell loss of 0.94/day, which was estimated from the average clone size simulated during the first week (Fig. S4D) and was similar to the expected replacement of stem cells according to the division cycle of stem cells (21.5 h). The generation of non-proliferating Paneth cells did not affect the neutral drift towards clonality.

After the first period of rapid clonal expansion the crypts remained in an oligoclonal state for relatively long and variable periods of time (Fig. S4A); the time required to observe the first monoclonal crypts varied from 15 to >200 days. Experimentally, the first monochromatic crypts are observed 2 weeks after labelling of stem cells with 75% of crypts becoming monoclonal after 2 months with some oligoclonal crypts still present after 30 weeks [31].

### Altering Signalling Pathways

**Figure 4:**
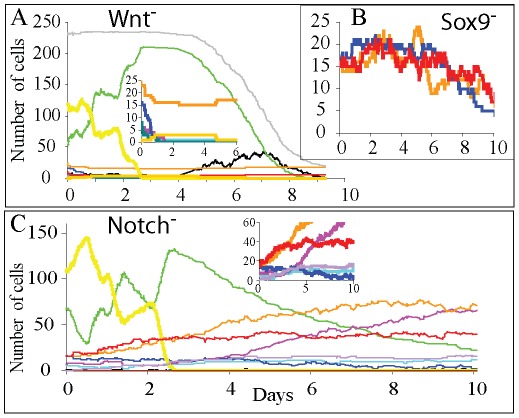
Blocking Wnt and Notch signalling in a steady state crypt. A) Blocking the Wnt signalling pathway halts cell proliferation and differentiation. Between days 2–3 all cells in the crypt differentiate into the enterocyte lineage with some aberrant Paneth cells scattered along the crypt. The colour code is as in Figure 3 with light orange lines representing aberrant mislocated Paneth cells in the crypt. Insert shows secretory and stem cells. B) Deletion of the Sox9 transcriptional factor of the Wnt signalling pathway blocks Paneth cell maturation which is coincident with a decline in the number of stem cells that co-localize with residual Paneth cells; C) Five or six days after blocking the Notch signalling pathway the entire proliferative absorptive progenitors compartment is populated exclusively by secretory cells**,** and while all secretory cell compartments expand, the number of stem cells decreases. Insert shows secretory and stem cells.

Our model is based upon reported observations [28] in which the absence of Wnt signalling halts stem cell proliferation forcing their differentiation into the enterocytic cell lineage (Fig. 1). In addition, we have modelled the effect of the inhibition of the Wnt signalling components Frizzle 5 receptor and Sox 9 and Math 1 transcriptional factors, by inhibiting death of Paneth cells migrating up the crypt, the maturation of progenitors into Paneth cells, and the differentiation of cells into the secretory cell lineage, respectively, as seen experimentally [16], [17], [50], [51]. Following inhibition of the Wnt signalling pathway, our model shows a complete block in proliferation by day 2 (Fig. 4A), which is the time needed for the proliferative absorptive progenitors to undertake committed divisions and differentiate into enterocytes. Thus, non-proliferative enterocytic cells were detected all along the simulated crypt including at the stem cell position with some Paneth cells mislocated along the crypt. After the first 2 days a progressive increase in apoptosis of the differentiated cells was detected leading to a marked reduction in the number of cells in the crypt by days 4–6. By day 8 the simulated crypts had less than 50 cells. These simulated results are consistent with the published experiments [28] (Fig. 4A) and the supplementary material includes a video (Video S3) of the simulation.

Paneth cells are important for maintaining the stem cell niche [19], [31]. In our model, we assumed that suppression of Sox9 blocked the maturation of the common goblet/Paneth cell progenitors into Paneth cells (Fig. 1) resulting in the complete disappearance of Paneth cells and their replacement by epithelial proliferative cells at the crypt base as previously reported [50]. The decrease in Paneth cells causes a local reduction of the predicted levels of Wnt signals and therefore a reduction in the number of stem cells which co-localize with the Paneth cells (Fig. 4B). These results are consistent with experimental observations [19], [31].

In our simulated crypt when Notch is blocked we assumed that the inhibition of the secretory fate requires contact with more than 80% of secretory cells, instead of the 50% required according to hypothesis III derived from the intermingled distribution of Paneth and stem cells in the crypt base (Figs. 2B, 2C). We also assumed that the maturation time of the common goblet-Paneth cell progenitor increases by a factor of three. Consequently the number of stem cells is markedly reduced while maximum Wnt signalling levels are maintained such that proliferative absorptive progenitors are not generated (Fig. 1). Consistent with recent findings [23], in the simulated crypt the proliferating cell compartment is replaced by secretory cells within 5 days, which are mostly goblet cells and common goblet-Paneth cell progenitors. The number of Paneth, enteroendocrine and Tuft cells also increases, while the number of stem cells decreases (Fig. 4C and video S4).

### Model Predictions

#### (1) Physical Interactions

**Figure 5:**
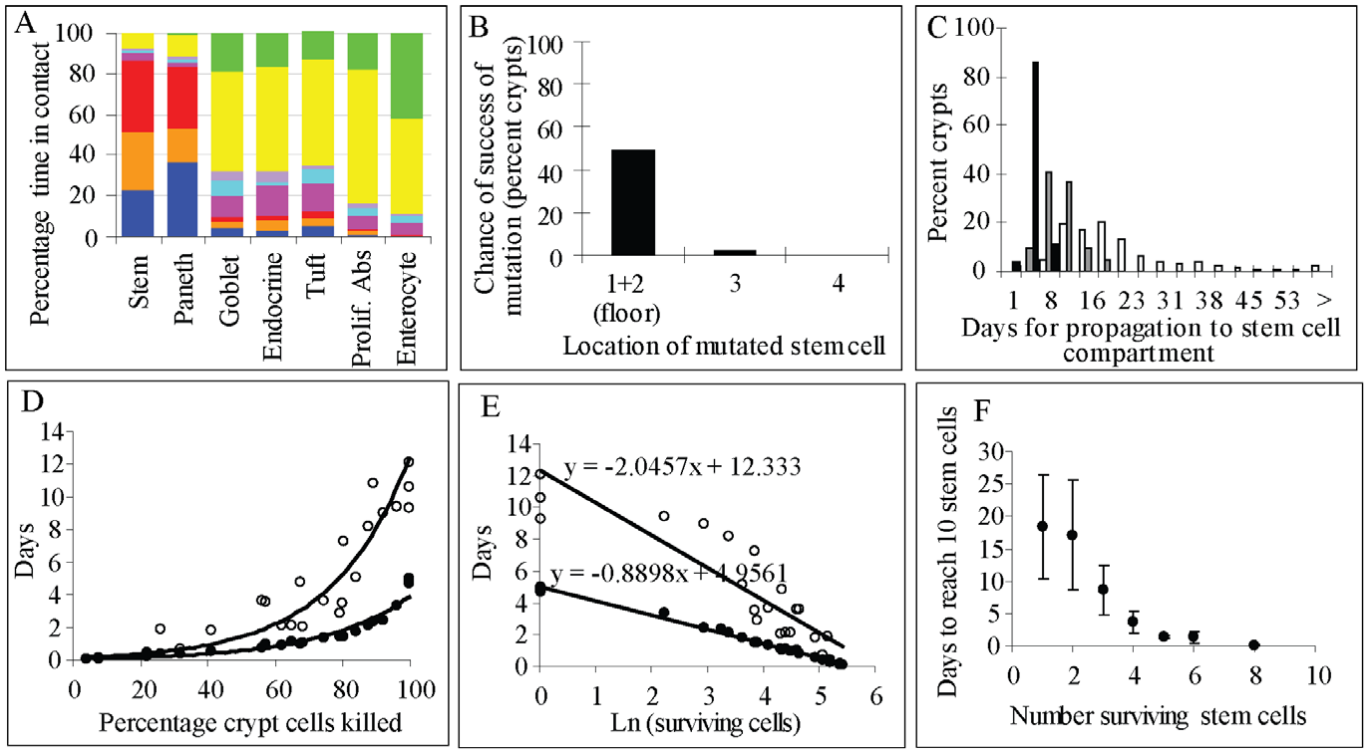
Model Predictions. A) Percentage of contact time between cell types in a mature crypt observed for 80 days. Colour code as in Figure 3. B) The chance of successful propagation of mutations affecting stem cells is ∼50% when the mutated cell is on the floor of the crypt and <2% for locations above. C) The time for the propagation of a mutation to the stem cell compartment is shorter when the mutation decreases the duration of the division cycle. Black columns show time for the propagation of mutated cells with 10-fold shorter division cycle, grey columns for 2-fold shorter, and white columns when division cycle is not affected. D) Time to replenish the crypt with the initial number of cells (•) and to recover the cellular structure of mature crypts after random killing of cells in the crypt (○). E) The numerical recovery rate of the injured crypt is 0.78 cells/day. The maturation rate is 0.34 cells/day. The time required to reach the original number of cells for an injured crypt that has lost half of its cells is approximately 0.9 days while the time required to reach a structural steady state is approximately 2 days. F) Time to replenish the bottom of the crypt with 10 Lgr5^hi^ cells after random killing of stem cells.

We have predicted the probability of physical interactions between cell types in the crypt by randomly sampling 500 times a simulated crypt in steady state for 80 days (Fig. 5A). In the simulated crypt, ∼70% of the cells in contact with stem cells were Paneth cells or their progenitors, while ∼20% were stem cells with the remaining cells being secretory cells and proliferative absorptive progenitors. These proportions of cells result from a non-random mixing process that is regulated by Notch signalling and increases the contact area between the two cell populations. It has been experimentally observed that 80% of the surface of stem cells is in contact with Paneth cells, with this physical interaction being important for the maintenance of the stem cells niche [19]. Goblet, enteroendocrine and Tuft cells as well as proliferative absorptive progenitors generated in the lower part of the crypt body eventually contact all the other cell types with a probability associated with the size of the crypt compartment. Mature enterocytes generated in the upper part of the crypt are unlikely to interact with stem or Paneth cells (Fig. 5A).

#### (2) Propagation of mutations

We have predicted the chance of successful propagation of mutations affecting stem cells by simulating 600 crypts with a mutated stem cell randomly located in the crypt bottom. A mutation was considered successful if it was propagated to all stem cells leading to the permanent mutation of the entire crypt. The success of mutations depended strongly on the position of the mutated cell. Mutations in stem cells located on the floor of the crypt (row 1 and 2) were successfully propagated in 50% of the cases while this decreased to 2% for mutated cells located in row 3 and to 0% in row 4 and above (Fig. 5B). Mutations involving acceleration of the division cycle up to 10 fold did not affect the probability of success, but markedly affected the propagation time of the mutation; ∼9 days when the cycle of the mutated cells was twice as fast as that of normal cells which was decreased to 3 days when it was 10 fold faster (Fig. 5C). The average propagation time of mutations that do not affect the duration of the division cycle was ∼20 days.

#### (3) Crypt damage

We have simulated the recovery process following crypt damage. The time taken to replenish the original number of cells and to reach steady state cell composition was estimated after cells were randomly killed in the crypt (Video S5). Steady state was reached when all cell compartments exhibited a size equivalent to those described for a normal crypt (Fig. S1). Recovery times increased exponentially as the proportion of killed cells in the crypt increased (Fig. 5D). On average the predicted numerical recovery of the crypt occurs at a rate of 0.78/day (Fig. 5E), which represents the number of cells generated by a surviving cell each day. The predicted time to recover the total number of cells in a damaged crypt with 10% cells surviving is approximately 3 days. It has been observed that 10 Gy X-irradiation leads to a 90% reduction in total crypt cell numbers during the first 2–3 days post-treatment followed by a rapid recovery period of 3–4 days in which the crypt reaches its original size [32]. Though model predictions are consistent with recovery times detected in irradiated crypts, we assume that crypt development following injury is the same as in homeostasis, which may lead to inaccurate results under different injury conditions. The predicted maturation rate after injuring the crypt is 0.34/day (Fig. 5E), which implies that the time required to recover and reach steady state for an injured crypt that has lost half of its cells is approximately 2 days.

#### (4) Behaviour of the Lgr5^hi^ and +4 stem cell pools

We assumed that both of these stem cell populations are part of the same stem cell pool but behave differently in response to different Wnt stimuli. When deleting Lgr5^hi^ stem cells, +4 stem cells can replace them in the crypt bottom. It has been shown that Lgr5^hi^ stem cells can be replenished from other crypt cell populations since markers of Lgr5 stem cells reappeared at the bottom of the crypt after their conditional deletion [10], [12], [15]. Additionally, in the absence of stem cells, proliferative absorptive progenitors progressively undergo intense apoptosis mirroring their limited self–renewal capacity and leading to the degeneration of the crypt [12]. We simulated the deletion of Lgr5^hi^ stem cells at the crypt bottom while leaving other cell compartments intact. Recovery time was arbitrarily established as the time required to reach a stage in development at which Paneth cells were intermingled with 10 Lgr5^hi^ stem cells. Recovery times depended on the initial number of surviving stem cells that repopulated the crypt bottom from +4 positions (Fig. 5F). Long recovery times accounted for initial low numbers of stem cells in the event they were located above Paneth cells, and require time in order to displace Paneth cells from the crypt bottom, which is a slow process as Paneth cells are static and long-lived.

## Discussion

We have approached modelling the crypt as a granular system, where cells represent the grains. Our model for the cell dynamical behaviour in the crypt is similar to that observed in the TETRIS model [52] or the Ising frustrated lattice-gas model subjected to gravity [53] and similar granular models [54], [55], with the external force here being cell growth. As in granular systems when the external force or cell growth is halted, cells come to rest. Despite the lack of a comprehensive understanding of cell dynamics in the crypt, we believe that its dynamic behaviour has similarities with that of granular or glassy systems. In our model, growth is homogenous in all directions. Increases in cell size creates a force translated mostly into upward migration because the crypt bottom is a rigid boundary. Figure S2B shows that the force derived from cell growth is sufficient to explain crypt cell dynamics.

The major findings of our study are, i) a demonstration of the usefulness of slow dynamic approaches to model the crypt from which a testable hypothesis has been derived for the localisation of Paneth cells to the crypt bottom without active forces but instead, as result of a slow structural relaxation phenomenon, and ii) that Notch signalling inhibits secretory fate if more than 50% of the cells in contact belong to the secretory lineage. In addition, other testable hypotheses that are supported by experimental evidences are that, (a) Wnt signalling depends on the local cell composition. Alterations of the stem cells niche composition such as the decrease of Paneth cells leads to a coincident decrease of stem cells that co-localize with the remaining Paneth cells and vice versa [19], (b) mature Paneth cells migrating up the crypt die by anoikis, which is experimentally explained by Eph/Ephrin signalling [37], (c) goblet and Paneth cells derive from a common goblet-Paneth cell progenitor [23] that is unrelated to enteroendocrine and Tuft cells [1], and (d) secretory cell progenitors do not proliferate as seen experimentally [35]. Comparisons between our model predictions and the available empirical evidence on crypt cellular structure, migration velocities, signalling alterations, and crypt development and recovery indicate that our hypotheses provide feasible mechanisms for cell proliferation, differentiation and kinetics in the small intestinal crypt.

Like previously described models our approach enables modelling of the dynamics of the crypt in steady state. In addition, our model allows the simulation of crypt generation from single stem cells, regeneration of the whole crypt from portions of it, and recovery of injured crypts.

The model of van Leeuwen et al. [24] describes a spatial gradient of extracellular Wnt factors along the crypt axis that determines position-dependent rates of cell proliferation, differentiation and death. The model of Buske et al. [26] refines the quantification of the spatial gradient as a function of the curvature of the crypt at the cell position. In our model Wnt signalling does not depend on the crypt external scaffold but it is quantified according to the local number of Paneth and stem cells as described in Equation 1. Alterations of the cell composition such as the decrease of Paneth cells leads to a reduction of Wnt signalling stimuli causing a coincident decrease of stem cells that co-localize with the remaining Paneth cells and vice versa. These predictions are consistent with recent observations [19]. Wnt signalling governs the fate of the stem cells progeny that will be Paneth and stem cells under the highest Wnt signalling otherwise stem cells give rise to secretory cells and proliferative absorptive progenitors. In both cases the decision between these two fates, secretory or stem/absorptive, depends on Notch signalling. We have found that Notch signalling inhibits secretory fate if more than 50% of the cells in contact belong to the secretory lineage and this criterion ensures both the maintenance of the correct proportion of stem cells at the base of the crypt and the generation of an appropriate number of all secretory cell types in the crypt body. Previous approaches to model Notch signalling did not consider stem cells and required the estimation of 3 parameters to describe a Notch threshold for secretory fate inhibition and two different levels of notch activation by Paneth and Goblet cells [26].

To date Paneth cells are assumed to be actively driven to the crypt base by forces derived from signalling processes [2], [26]. However, the presence of intermingling proliferating stem cells amongst Paneth cells [19] whose descendents are migrating upwards complicates the understanding of the downward migration of Paneth cells. Our modelling approach does not include active forces to account for downward migration to the crypt bottom of goblet/Paneth progenitors and/or mature Paneth cells. Instead, the location of Paneth or Paneth progenitors intermingling with stem cells at the crypt bottom is driven by the requirements for differentiation according to Notch signalling (Figs. 2B–C) and by the slow structural relaxation approach in which non-proliferating Paneth cells once located below stem cells remain there (Figure S3). Another distinctive feature is that our model does not describe an external structural scaffold, which is covered by a monolayer of cells. Cells organize themselves in a three dimensional cylindrical structure whose size depends on the number of cells. Moreover, the rings of the spiral and helix change dynamically to accommodate the increasing size of growing cells and/or decreasing size of dying cells. Previous approaches based on rigid lattices [56], [57] or on Voronoi tessellations [25]are constrained to fixed shapes the size of which cannot be changed dynamically. Furthermore, in our model, the size of the entire crypt is dynamic and able to respond to changes in cell production demanded by adjacent villi, It can therefore be extended to the intestinal mucosa by combining crypts of variable size according to cell production demanded by neighbouring villi.

## Methods

### Cell Growth

Cells are modelled as regular convex shapes in two dimensions. For proliferating cells, the cell surface, *S*, grows at a constant rate, *k =  S_ini_/D_c_*, reaching twice its initial size, *S_ini_*, during the duration of the division cycle, *D_c_*. For cells undertaking differentiation, the cell surface grows to reach a maximum cellular size, *S_max_*, according to:
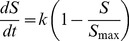
(3)


where *k* is the growth rate determined at birth as described above.

The time to reach *S_max_* is the maturation time. During maturation time goblet-Paneth cell common progenitors (Fig. 1) remain undifferentiated. Cells reaching the end of their lifespan undergo apoptosis and progressively detach from the mesenchyme. During apoptosis the crypt surface covered by the cell decreases at a constant rate estimated as *h*  =  *S_max_*/(0.1*Lifespan).

The cell surface area is assumed to have a normal distribution. Estimations of ca. 10 and 13 µm for the mean surface area of proliferating and non-proliferating cells, respectively, and of 0.05 for their coefficient of variation (CV  =  standard deviation/mean) were empirically determined. Both *S_ini_* and *S_max_* are randomly generated at birth time.

### Cell Division Cycle and Lifespan

The duration of the division cycle, *D_c_*, has been reported to decrease as cells escalate up the crypt [32]. An average cell cycle length equal to 21.5 h has been observed for Lgr5^hi^ stem cells [13] while +4 stem cells have been reported to divide once a day [58], [59] and the measured shortest cycle length of a proliferative progenitor is of 9.7 hours [32]. We assumed that *D_c_* decreases as proliferative absorptive progenitors undertake successive divisions during their migration up the crypt. *D_c_* is assumed to have a gamma distribution and its parameters are estimated by the method of moments from the mean value and CV. The estimation of CV was equal to 0.144 according to published data and no association between the value of CV and the duration of the cell cycle has been observed [32]. In order to find the relationship between the mean value, 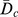, and the number of undertaken divisions, *N_d_*, we ran our model to simulate *N_d_* values at each crypt location and correlated them with the mean value for the duration of the cell cycle, 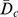, at each crypt location as previously observe [13], [32]. The relationship was as follows (Fig. S5A):
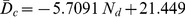
(4)


For stem cells, *N_d_* is fixed to 0. Data shows a lack of dependence for proliferative progenitors that undertake more than 2 division cycles; hence the maximum value of *N_d_* is fixed to 2. Observed and estimated cell cycle durations according to cell position in the crypt were consistent (Fig. S5B).

Lifespan was modelled as a gamma distributed random variable for all cell lineages. Parameters of the distributions were estimated from empirically derived mean values and CV. Longevity of Paneth cells had a expected value equal to 54 days based on published studies [60]. The mean values for the lifespan of goblet and endocrine cells were equal to 5 and 23 days, respectively, as previously reported [32] while for enterocytes the mean value was estimated as 5 days from published results on the loss of all intestinal epithelial cells after blocking Wnt signalling [28] The CV of these variables was assumed to be equal to 0.144 as reported by Wright [32] for other cell cycle times.

### Number of Divisions Undertaken by Proliferative Absorptive Progenitors

As previously proposed [61], the total number of proliferating cells is the sum of the expected number of cells in each proliferative compartment. The first proliferative compartment contains Lgr5^hi^ stem cells, named *Lgr5^hi^*. The second compartment contains +4 stem cells, named *Stem4*, that receive an influx of cells from the previous compartment and provides an efflux of cells to the following compartment, *T_1_*, of proliferative progenitors in first division, the descendents of these are proliferative progenitors in second division, *T_2_*, and so on until the *n-th* division, *T_n_*. The flux of cells between the first three compartments cannot be calculated straightforwardly from generation rates because part of the newly generated cells in these compartments will be non-proliferative Paneth, goblet and enteroendocrine cells with their replacement rate depending on the velocity of cell migration and rate of apoptosis at the bottom of the crypt. The expected number of cells in the compartments of proliferative absorptive progenitors, *T_i_* for *i* >1, can be estimated as the efflux of cells coming from the previous compartment
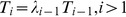
(5)


Where *λ*_i−1_ is the growth rate of the cell population in the previous compartment estimated as:
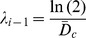
(6)


*D_c_* estimated from equation (4).

The following expression describes the number of proliferating cells, *N_prolif_*, in the crypt:
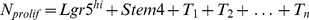
(7)


As explained above *λ_i_*  =  *λ_2_* for *i* >1, thus the number of committed divisions, *n*, to be undertaken by proliferative absorptive progenitors can be estimated from equation (7) as follows:

Then
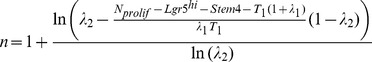
(8)


From published data [32], [44], it can be estimated that about 73% of the cells in a mature crypt are proliferating cells (Fig. S5C). We simulated crypts with sizes between 100 and 400 cells and solved equation (8) with the values obtained for the number of cells in the Lgr5^hi^, *+*4 stem cells and *T*_1_ compartments (Fig. S5D). The estimated number of divisions to be undertaken by proliferative progenitors, *n*, varied from ca. 5.4 to 6.7 (Fig. S5D), with a mean value of 6, which is the value used in the model.

### Organization of the Three Dimensional Crypt

The bottom of the crypt is modelled as a three dimensional spiral followed by the crypt body, which is a three dimensional helix, constructed from single cells organized in a one-dimensional chain. The position of any cell in the spiral or helix is determined by the polar coordinates *r* and *θ* and the vertical coordinate z.

The value for the polar coordinate, *r_i_,* of the *i-th* cell is estimated as
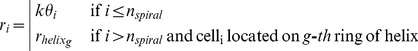
(9)


The value for *θ_i_*, the polar angle of the *i-th* cell is equal to
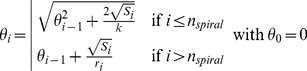
(10)where the square root of *S_i_* represents the width of the *i-th* cell.

The third coordinate *z_i_* is calculated as follows:
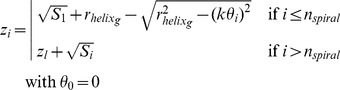
(11)where *l* is the index of the closest cell to the *i-th* cell from the ring immediately below.

The initial values for 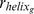, *k* and *n_spiral_* in equations (9), (10) and (11) are estimated according to the relationships between morphometric measurements of the crypt, cell sizes, number of proliferative cells and total cells in the crypt from published data of small and large intestinal mucosa of humans [44] and mice [32]. Based on these relationships, the total number of cells, *ncells*, is the only input parameter needed to build the crypt.

The relationship between the total number of cells and the circumference of the crypt is as follows (Fig. S5E):
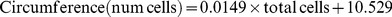
(12)From the same dataset we could estimate that 73% of the cells in a mature crypt are proliferating cells (Fig. S5C). Hence the initial value for the radius of any ring in the helix, i.e. for 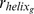 in equations (9) and (11), is equal to:
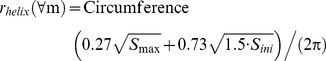
(13)where Smax is the surface area of mature Paneth cells and (1.5 Sini) is the average surface area of proliferating cells.

Assuming that the bottom of the crypt is a semi-sphere, its surface can be estimated as:
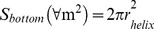
(14)


Knowing that there are aproximately 20 Paneth cells [32] and 14 Lgr5+^hi^ stem cells [31] at the base of the crypt, and assuming that this proportion holds for any number of cells in the crypt, the initial estimation of the number of cells covering the surface of the spiral will be:
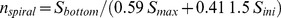
(15)


The first *n_spiral_* cells of the one dimensional chain of cells will be located on the spiral and the initial value for the length of the spiral can be approximated as
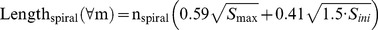
(16)


Since the polar coordinate *r* for the last cell in the spiral should be equal to the initial radius of the helix, the initial value for *k* in equations (9), (10) and (11) is equal to:
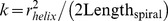
(17)


Cells divide, grow, migrate and undergo apoptosis. These processes affect the cell size. The rings of the crypt are flexible to accommodate changes in cell size (Fig. S2A). To achieve this, the values for *k* and 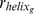
from equations (9), (10) and (11) are calculated for the *m-th* time step of a simulation as follows:
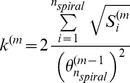
(18)and
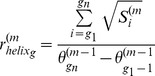
(19)where {g1 .. gn} is the set of index in the g-th ring of the helix.

In this way changes in cell size affects the radius of the spiral and helix. The rings of the crypt expand and shrink to accommodate these changes while the angular position of the cells is virtually unaffected, which minimize lateral swings of cells (Fig. S2A,C).

We assumed that cells located on the spiral form the crypt bottom cap, which is characterized by the highest levels of Wnt signalling and contains Lgr5^hi^ stem cells and Paneth cells (Fig. 1). Figure S5F shows the number of Lgr5^hi^ stem cells, Paneth cells, and proliferative absorptive progenitors generated a day and the diameter and length of simulated crypts of several sizes between 100 and 400 cells.

### Spatial Organization of Organoids During Development from One Single Cell

When modelling crypt development from a single cell, crypts are initially spheroids with a central lumen. The radius of the sphere containing *n* cells is estimated as:
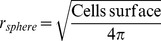
(20)where



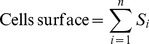
(21)When the number of cells, *n*, reaches the target crypt size, the elongation of the crypt is modelled by the progressive decrease of the radius of the sphere that becomes a closed cylinder. The radius of the sphere decreases to the value of *r_helix_* in equation (13) in about one day time. During the elongation time the crypt structure is still closed and the parameters for the bottom and top spirals are estimated according to equations (15–17).

### Cell Dynamics

Growing cells expand in all directions and this expansion affects its position and the position of the other cells when the crypt is reorganized at each time interval. The size of the rings of the spiral changes dynamically to accommodate cell size increases in the horizontal direction, i.e. perpendicular to the crypt-villus axis. The increase in cell size in the parallel direction to the crypt-villus axis creates a force translated mostly as upward migration because the crypt bottom is a rigid boundary and we do not consider the possibility of cell compression. Downward motion could eventually be observed involving very short trajectories when cells located in lower positions undergo apoptosis (Fig. S2C). Therefore, the functional form for the instantaneous vertical velocity of cell *j*, *v_i_*:
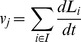
(22)


Where *I* is the set of indices of those cells located in the vertical axis below cell *j* and *L*_i_ is the length in the vertical axis of cell *i*. Figure S2B shows the average vertical cell velocities calculated by simulation for each ring in the crypt.

After division a new cell has to be located in the neighbourhood of the original cell. The division event does not imply any increase of cell surface and thus its impact on cell dynamics can be neglected. In practice, after division the one dimensional chain used to build the three dimensional helix has to be reorganized to assign an index to the new cell. The new cell takes either the index of any apoptotic cell in contact, which is deleted, or otherwise it takes the index of the closest cell in the ring immediately above, position at which the new cell is already protruding. The vertical adjustment of indices spreads upwards along the crypt until the cell in the last ring, which is usually protruding out of the crypt, moves from the crypt to the adjacent villus. Newly generated cells in the first spiral ring forming the floor of the crypt are located laterally or vertically with equal probability. Therefore the reassignment of indices locates cells in their actual ring position after displacements due to growth.

### Simulation

A Visual Basic Excel add-in has been written in Visual Basic to simulate the crypt. At simulation times, Wnt activity, size, position and age of all cells are updated. Time steps are variable.

## Supporting Information

Figure S1 **Simulated crypt in steady state.** A) The estimated proportion of different epithelial cell types at different position from the bottom of the crypt obtained from 250 simulated crypts. The predicted number of goblet, Tuft and enteroendocrine cells decreases as their position up the crypt increases. Apoptotic cells are detected at the bottom of the crypt in the stem cell area. B) Experimental and simulated proportions of proliferating cells according to their position in the crypt. The proportion of simulated proliferating cells (black symbols) decreases as their position in the crypt increases, consistent with observed data (red symbols) [26].
(TIF)

Figure S2 **Slow dynamics in steady state.** A) Average diameters (µm) and standard deviation for each ring in the crypt. The size of rings changes dynamically to accommodate increases and decreases the size of cells; B) Cell migration velocities increase with increasing position up the crypt. Simulated velocities (black symbols) are consistent with measured migration velocities (red symbols) [3]. C) Representation of cell migration trajectories in the 2 dimensional sheet of the crypt opened longitudinally. Symbols mark the initial position of the cell or, its conversion into another cell type. Colour code is as in Figure S1.
(TIF)

Figure S3 **Slow dynamics organization.** As shown for a longitudinally opened crypt with proliferating (blue) and no proliferating cells (orange), which starts with 7 proliferative cells below 33 non-proliferative cells. Proliferative cells divide generating two daughter cells, one proliferative and one non-proliferative cell that are randomly chosen. Division cycle is a gamma distributed variable with a mean value of 21 h and coefficient of variation of 0.144. After 4 days proliferative cells gain positions upwards from the crypt bottom. By day 25 they reside above non-proliferative cells.
(TIF)

Figure S4 **Clonal evolution and neutral drift dynamics.** A) The number of clones decreases rapidly in the first week from 14 clones containing 1 Lgr5^hi^ stem cell to 2–3 clones. The persistence of oligoclonal crypts is highly variable. The results show three different simulations (black, dark and light gray symbols). B) Distribution of Paneth cells (diamonds) and Lgr5^hi^ stem cells (circles) during progression of stem cells to monoclonality. Clonal populations of cells are shown as the same colour. C) Cumulative clone size distribution or probability of finding a clone with more than *n* Lgr5^hi^ stem cells. The lines show predictions for days 1 (lower line), 2, 3, 5, 7, 9 and 11 (upper line) marked with different colours using the scaling function characteristic of neutral drift dynamics [31], [49] with the estimated stem cell loss rate calculated from data shown in Figure S4D. The points show the simulated results from days 1 to 11. Scaling behaviour is consistent with the model hypothesis of equipotency of all Lgr5^hi^ stem cells in the crypt. D) Average number of Lgr5^hi^ cells within surviving clones increases rapidly in the first week following the decrease in the number of clones. From the first week of simulation the stem cell replacement rate is estimated as 0.98 cells/day. Inset shows that during the first week the average clone size follows a square root time dependency as predicted [31] for equipotent stem cells. Results shown are from three different simulations (black, dark and light gray symbols).
(TIF)

Figure S5 **Support material for model development and parameter estimation.** A) Relationship between the average duration of the cell division cycle at several crypt positions according to published data [32] and the average number of divisions at each crypt position simulated by the model. B) Comparison between the observed (red) and simulated (black) duration of the cell division cycle at several crypt positions using published data [32]. C) Relationship between the total number of cells and the number of proliferating cells. Published data is from human small intestinal mucosa (black points) [44] and murine small (pink) and large (green) intestinal mucosa [32]. The line shows the fitted equations. D) Number of committed divisions to be undertaken by proliferative absorptive progenitors as a function of the total number of cells of the crypt. E) Relationship between the total number of cells and the number of cells on the crypt circumference from published data is as in Figure 5C. F) Number of Paneth cells (orange) Lgr5^hi^ stem cells (blue) proliferative absortive progenitors (yellow) and generated cells per day (purple) and the length (white) and diameter (gray) of simulated crypts of several sizes. Measurements are expressed as number of cells.
(TIF)

Video S1 **Generation of a crypt from a single stem cell and its development over 40 days.**
(MOV)

Video S2 **Clonal expansion from 14 equipotent Lgr5^hi^ stem cells in a simulated crypt comprising all cell lineages over 53 days.**
(MOV)

Video S3 **Simulation of cell proliferation and differentiation in the crypt after inhibiting the Wnt signalling pathway.**
(MOV)

Video S4 **Simulation of cell proliferation and differentiation in the crypt after blocking Notch signalling.**
(MOV)

Video S5 **Simulation of the recovery of the crypt following the random killing of 90% of the crypt cells.**
(MOV)
