# Supplementary figures and images for: Modelling the Spatio-Temporal Cell Dynamics Reveals Novel Insights on Cell Differentiation and Proliferation in the Small Intestinal Crypt
Source: PLoS One. 2012 May 18;7(5):e37115. doi: 10.1371/journal.pone.0037115 (PMC3356410; doi:10.1371/journal.pone.0037115)

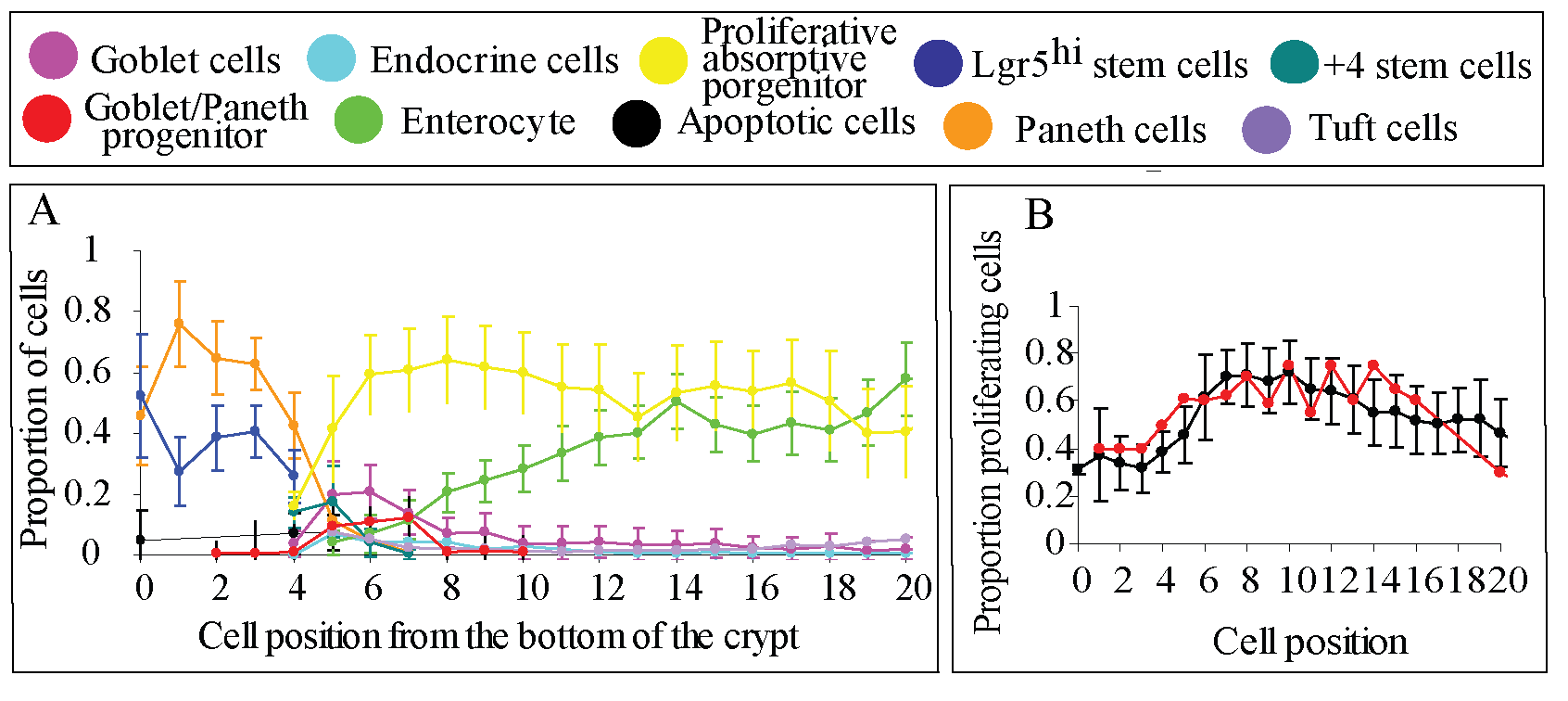

Supplement: Figure S1 — Simulated crypt in steady state. A) The estimated proportion of different epithelial cell types at different position from the bottom of the crypt obtained from 250 simulated crypts. The predicted number of goblet, Tuft and enteroendocrine cells decreases as their position up the crypt increases. Apoptotic cells are detected at the bottom of the crypt in the stem cell area. B) Experimental and simulated proportions of proliferating cells according to their position in the crypt. The proportion of simulated proliferating cells (black symbols) decreases as their position in the crypt increases, consistent with observed data (red symbols) [26]. (TIF) [file pone.0037115.s001.tif]

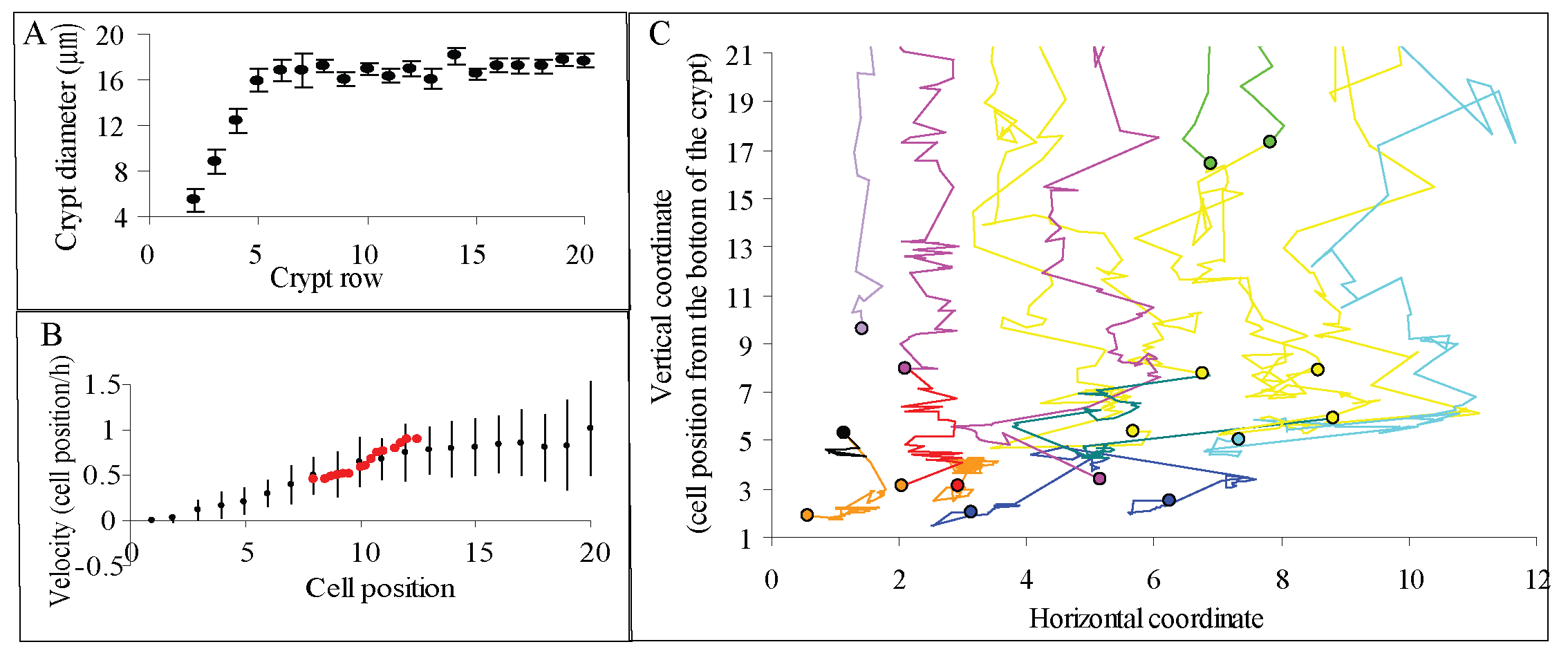

Supplement: Figure S2 — Slow dynamics in steady state. A) Average diameters (µm) and standard deviation for each ring in the crypt. The size of rings changes dynamically to accommodate increases and decreases the size of cells; B) Cell migration velocities increase with increasing position up the crypt. Simulated velocities (black symbols) are consistent with measured migration velocities (red symbols) [3]. C) Representation of cell migration trajectories in the 2 dimensional sheet of the crypt opened longitudinally. Symbols mark the initial position of the cell or, its conversion into another cell type. Colour code is as in Figure S1. (TIF) [file pone.0037115.s002.tif]

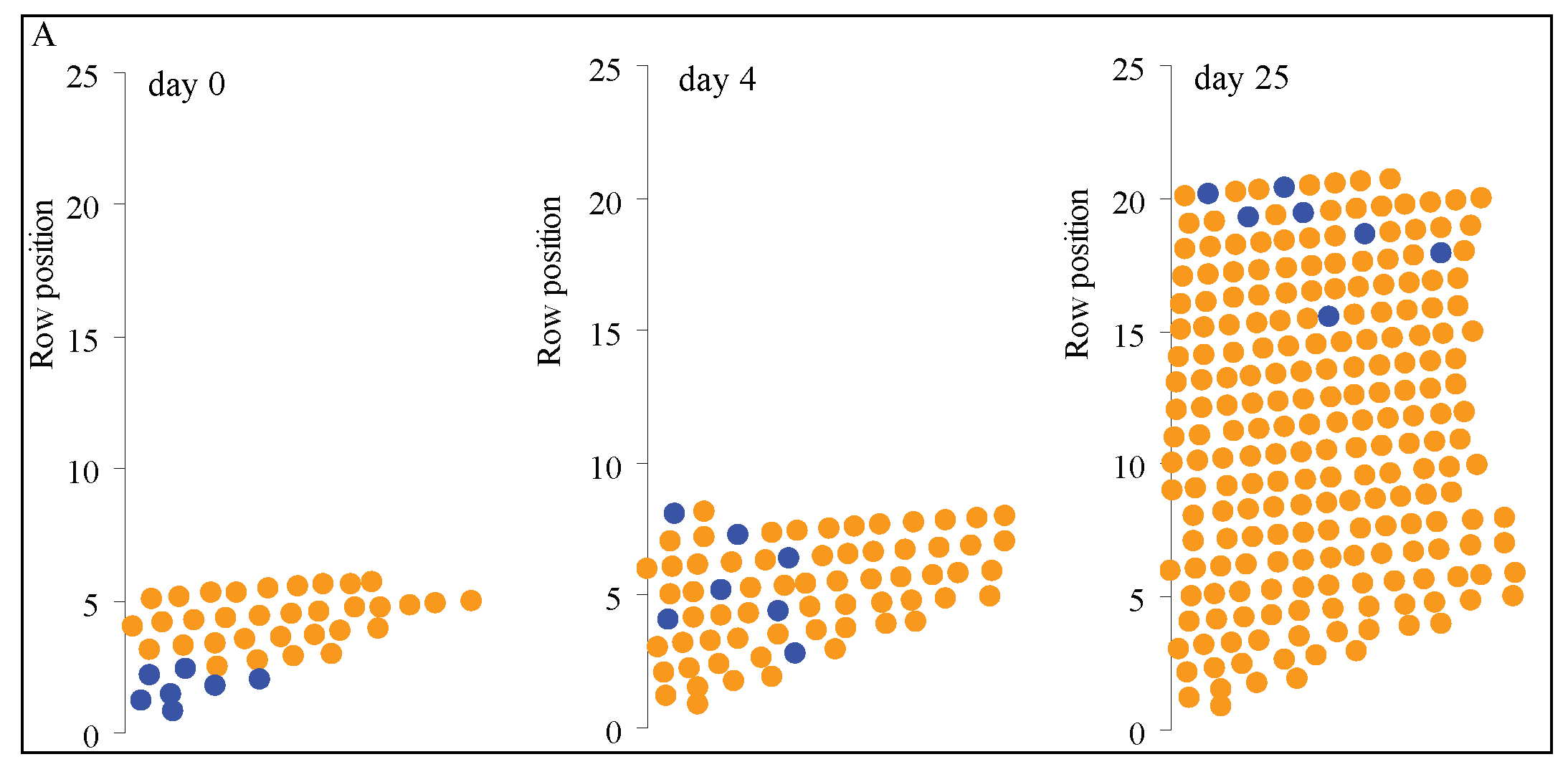

Supplement: Figure S3 — Slow dynamics organization. As shown for a longitudinally opened crypt with proliferating (blue) and no proliferating cells (orange), which starts with 7 proliferative cells below 33 non-proliferative cells. Proliferative cells divide generating two daughter cells, one proliferative and one non-proliferative cell that are randomly chosen. Division cycle is a gamma distributed variable with a mean value of 21 h and coefficient of variation of 0.144. After 4 days proliferative cells gain positions upwards from the crypt bottom. By day 25 they reside above non-proliferative cells. (TIF) [file pone.0037115.s003.tif]

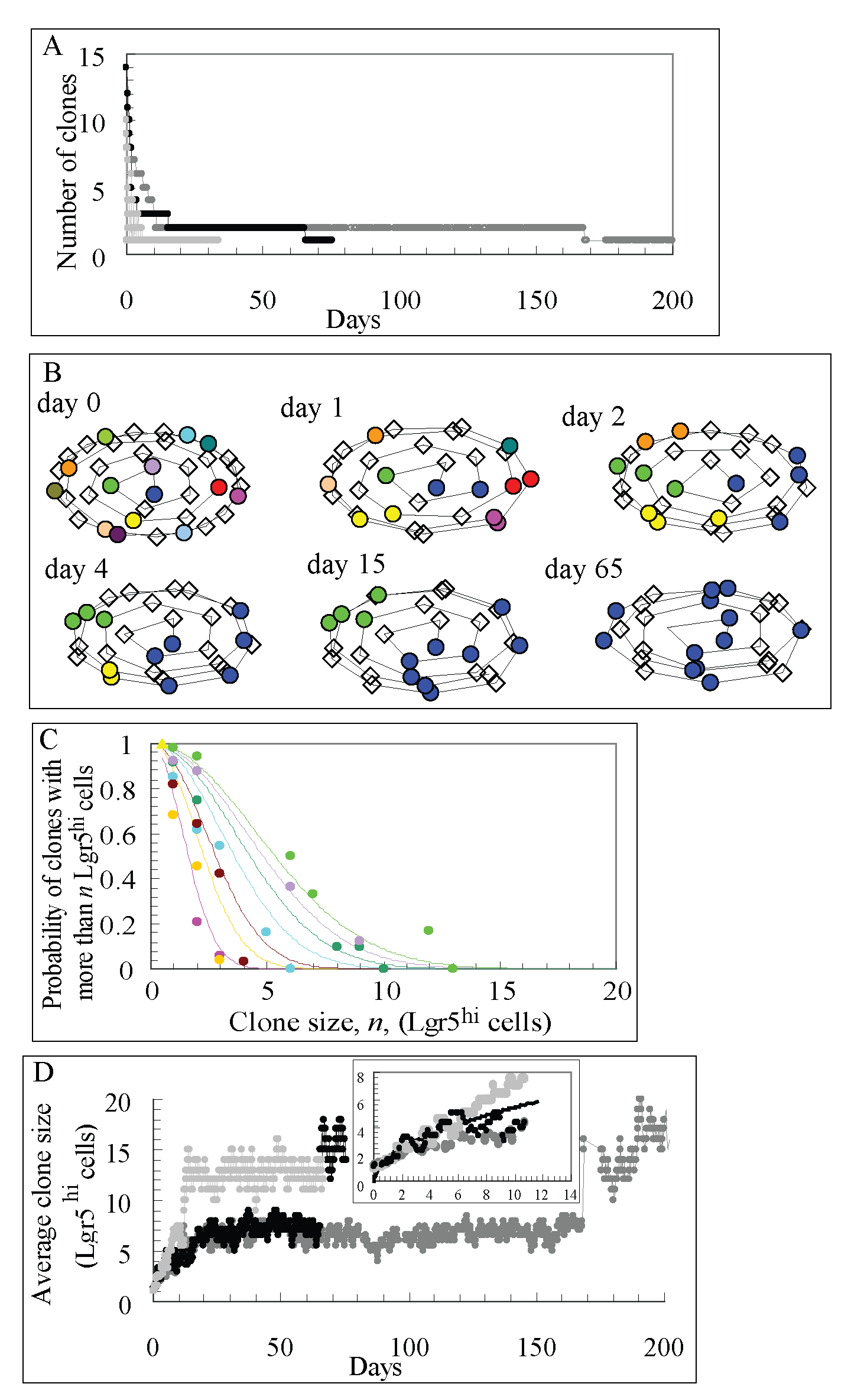

Supplement: Figure S4 — Clonal evolution and neutral drift dynamics. A) The number of clones decreases rapidly in the first week from 14 clones containing 1 Lgr5hi stem cell to 2–3 clones. The persistence of oligoclonal crypts is highly variable. The results show three different simulations (black, dark and light gray symbols). B) Distribution of Paneth cells (diamonds) and Lgr5hi stem cells (circles) during progression of stem cells to monoclonality. Clonal populations of cells are shown as the same colour. C) Cumulative clone size distribution or probability of finding a clone with more than n Lgr5hi stem cells. The lines show predictions for days 1 (lower line), 2, 3, 5, 7, 9 and 11 (upper line) marked with different colours using the scaling function characteristic of neutral drift dynamics [31], [49] with the estimated stem cell loss rate calculated from data shown in Figure S4D. The points show the simulated results from days 1 to 11. Scaling behaviour is consistent with the model hypothesis of equipotency of all Lgr5hi stem cells in the crypt. D) Average number of Lgr5hi cells within surviving clones increases rapidly in the first week following the decrease in the number of clones. From the first week of simulation the stem cell replacement rate is estimated as 0.98 cells/day. Inset shows that during the first week the average clone size follows a square root time dependency as predicted [31] for equipotent stem cells. Results shown are from three different simulations (black, dark and light gray symbols). (TIF) [file pone.0037115.s004.tif]

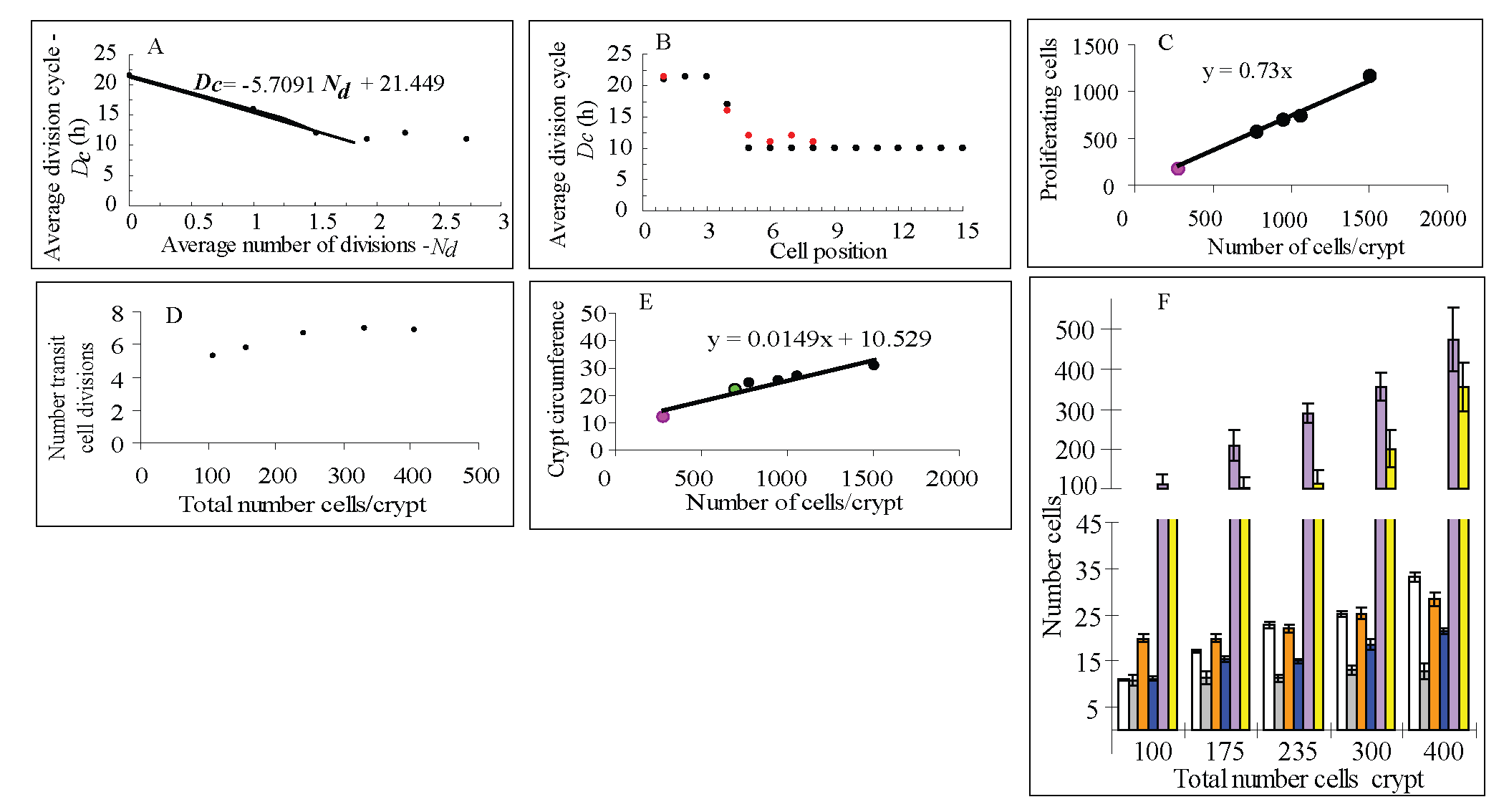

Supplement: Figure S5 — Support material for model development and parameter estimation. A) Relationship between the average duration of the cell division cycle at several crypt positions according to published data [32] and the average number of divisions at each crypt position simulated by the model. B) Comparison between the observed (red) and simulated (black) duration of the cell division cycle at several crypt positions using published data [32]. C) Relationship between the total number of cells and the number of proliferating cells. Published data is from human small intestinal mucosa (black points) [44] and murine small (pink) and large (green) intestinal mucosa [32]. The line shows the fitted equations. D) Number of committed divisions to be undertaken by proliferative absorptive progenitors as a function of the total number of cells of the crypt. E) Relationship between the total number of cells and the number of cells on the crypt circumference from published data is as in Figure 5C. F) Number of Paneth cells (orange) Lgr5hi stem cells (blue) proliferative absortive progenitors (yellow) and generated cells per day (purple) and the length (white) and diameter (gray) of simulated crypts of several sizes. Measurements are expressed as number of cells. (TIF) [file pone.0037115.s005.tif]
